# Environmental measures to improve pedestrian safety in low- and middle-income countries: a scoping review

**DOI:** 10.1177/17579759241241513

**Published:** 2024-05-08

**Authors:** Saidou Sabi Boun, Ronaldo Janvier, Rose Eveyoung Jean Marc, Peterline Paul, Rachel Senat, Joseph Adrien Emmanuel Demes, Guillaume Burigusa, Sarah Chaput, Pierre Maurice, Thomas Druetz

**Affiliations:** 1 Department of Social and Preventive Medicine, School of Public Health, University of Montreal, QC, Canada; 2 School of International Development and Global Studies, University of Ottawa, Ottawa, ON, Canada; 3 School of Human Sciences, State University of Haiti, Port-au-Prince, Haiti; 4 School of Medicine and Pharmacy, State University of Haiti, Port-au-Prince, Haiti; 5 Institut national de santé publique du Québec, Quebec, QC, Canada; 6 Réseau francophone international pour la promotion de la santé, Montreal, QC, Canada; 7 Centre de recherche en santé publique, Montreal, QC, Canada; 8 Department of Tropical Medicine, School of Public Health and Tropical Medicine, Tulane University, USA

**Keywords:** vehicle–pedestrian accidents, road injuries, road crashes, low- and middle-income countries, pedestrian victims

## Abstract

**Objectives:**

This scoping study aims to identify environmental road safety measures implemented in low- and middle-income countries (LMICs) to reduce pedestrian injuries from collisions with motor vehicles.

**Methods:**

This review followed Arksey and O’Malley’s approach and reported results using the PRISMA-SCR 2018 checklist. A literature review was conducted in Medline, Google Scholar, and the Transport Research International Documentation database using keyword-derived medical subject heading terms. A total of 14 articles met the pre-established inclusion criteria and were analyzed using a data extraction matrix. The findings were categorized methodically into three prominent themes: (1) methods for reducing pedestrian exposure, (2) traffic calming strategies, and (3) measures for enhancing pedestrian visibility.

**Results:**

Traffic calming strategies, including vehicular speed reduction, roadway contraction, and vertical and horizontal diversionary tactics, emerged as the most effective interventions for reducing pedestrian injuries within LMICs. Conversely, interventions geared towards minimizing pedestrian exposure, such as zebra crossings, crosswalks controlled by traffic signals, underpasses, or overpasses, often produced minimal effects, and occasionally exacerbated the risk of pedestrian accidents. Lack of pedestrian visibility due to density of street vendors and parked vehicles was associated with a higher risk of injuries, while billboards impaired drivers’ attention and increased the likelihood of collisions with pedestrians.

**Discussion:**

In LMICs, the effectiveness of environmental measures in reducing vehicle–pedestrian crashes varies widely. In the face of resource constraints, implementing interventions for pedestrian safety in LMICs necessitates careful prioritization and consideration of the local context.

## Introduction

The number of deaths from road accidents was estimated at 1.19 million in 2021, which corresponds to a rate of 15 per 100,000 habitants (1). Road traffic injuries were the 12th leading cause of death for all ages and the first leading cause of death worldwide for people aged 5–29 years. The mortality rate relative to the world’s population has been decreasing steadily since 2010, when it reached a peak of 18 per 100,000 population. Considering that the number of motor vehicles on the road increased by 160% over that period, the reduction in death per 100,000 vehicles, i.e., from 79 to 41, becomes even more important.

These figures are averages that mask inequalities in progress from one region and country to another. There is a disparity between high-income countries (HICs) and low- and middle-income countries (LMICs). Overall, it appears that the risk of death is linked to a country’s income level, with a mortality rate nearly three times higher in low-income countries than in HICs, with 21 versus 8 deaths per 100,000 inhabitants, respectively (1). An astonishing 92% of road accidents worldwide occur in LMICs, even though they account for only 72% of motor vehicles globally. Given these conditions, the World Health Organization (WHO) warns that Target 3.6 of its Sustainable Development Goals (i.e., reducing by half the number of deaths and injuries worldwide due to road accidents) may be missed by 2030, and calls for a ‘paradigm shift in leadership, commitment, investment and action (1,2).’

Pedestrians are among the most vulnerable road users, accounting for 23% of road deaths (1,2). This proportion is even higher in LMICs, reaching 27% in Africa. There are several reasons for the excess risk to pedestrians in LMICs: the rapid growth of urbanization and motorization; the mix of traffic involving trucks, cars, cyclists and pedestrians; and, above all, the lack of separation between different road users, which disproportionately increases pedestrian risk, exacerbating existing health inequities (3,4). Roads are generally built to satisfy the needs of motorists foremost, neglecting the needs of pedestrians, whose lack of physical protection makes them much more vulnerable to injury in road accidents. Pedestrians also tend to comprise children and the elderly, whose cognitive and psychomotor development and physical abilities, respectively, further add to their risk of injury (5,6).

Vehicle–pedestrian injuries (VPI) include all events where at least one pedestrian is injured by a motor vehicle in motion. In addition to their effects on public health, road accidents hamper the development of LMICs: they lead to work and school interruptions, loss of productivity, and very high health and socioeconomic costs for victims, families, and countries. A 2017 study showed that a 50% reduction in road accident injuries and deaths could generate an additional income stream of 7–22% of GDP in some LMICs (7).

Despite this burden, it remains unclear which policies and interventions are effective in reducing VPIs in LMICs. Some literature reviews have explored this topic, but were either limited to HICs or were not focused on pedestrian victims (8–11), and there are several urgent reasons to review literature specifically focused on VPIs in LMICs. Firstly, the contexts differ greatly between HICs and LMICs, and, arguably, even between LMICs. While income level is associated with stratification of the burden between countries, it is reasonable to assume that the effectiveness of interventions may also vary according to countries’ wealth levels or other sociocultural characteristics (11). Secondly, while many different policies can reduce road accidents, their effectiveness in reducing morbidity and mortality varies by the type of user. This review focuses strictly on interventions to reduce the risk of pedestrians – the most vulnerable of road users. In the same vein, it was decided to limit the literature review to studies that examined environmental measures to reduce VPIs, i.e., those interventions that act on the built environment and are described as ‘passive’ because they require no effort or participation on the part of the individual being protected (12). By adopting a systemic approach, environmental measures are highly successful since they bring about lasting changes that act on all individuals, whatever their age, sex, state of health, literacy, behavior, or socioeconomic level (13,14). Finally, these measures contribute to reducing health inequities, which is of particular interest for LMICs (12,15).

This scoping review aims to understand the nature and extent of the understudied phenomenon of VPIs and the environmental interventions implemented in LMICs to reduce them. By filling this important gap, we hope to help inform national authorities of the best strategies for improving pedestrian road safety.

## Methods

### Search strategy

The review followed the approach advocated by Arksey and O’Malley (16) for conducting scoping reviews, which comprises five steps: (1) formulation of the research question; (2) identification of relevant studies; (3) selection of studies according to inclusion and exclusion criteria; (4) extraction and mapping of data categorized according to key findings; and (5) reporting of results. The PRISMA-SCR checklist (Preferred Reporting Items for Systematic Reviews and Meta-Analysis Extension for Scoping Reviews) was used to report, filter, and communicate our results (Appendix) (17). The protocol was not published.

The first step consisted of identifying the three key concepts that guided this literature search: ‘road accidents,’ ‘pedestrians,’ and ‘environmental meas-ures’ (see Appendix 1). A limited search was performed in Google Scholar to identify additional terms and synonyms used in the literature to refer to these concepts. The complete list was discussed among the authors to seek additional suggestions, and derived MESH (Medical Subject Heading) terms were identified.

In the second step, all identified keywords and MESH terms were searched in three databases: Medline, Google Scholar, and the Transport Research International Documentation database. There was no time limit (the search was conducted in March 2022). Boolean operators (*and*, *or*) combined the keywords and their synonyms, and the truncation sign * was used at the end of the keywords when appropriate. The following search equation was generated:

((((road* *or* highway* *or* traffic) *and* (safety *or* security *or* accident* *or* injury* *or* crash* *or* collision*)) *and* pedestrian*) *or* (pedestrian-vehicle collision* *or* pedestrian-vehicle accident* *or* pedestrian-vehicle crash* *or* vehicle-pedestrian collision* *or* vehicle-pedestrian accident* *or* vehicle-pedestrian crash*)) *and* (urban* *or* town* *or* city *or* cities *or* built environment *or* city planning *or* road* *or* pedestrian* crossing* *or* street*)

This search equation was replicated across the three search engines. Although the search terms were all in English, publications in French were also considered.

### Inclusion/exclusion criteria

**Figure 1.**
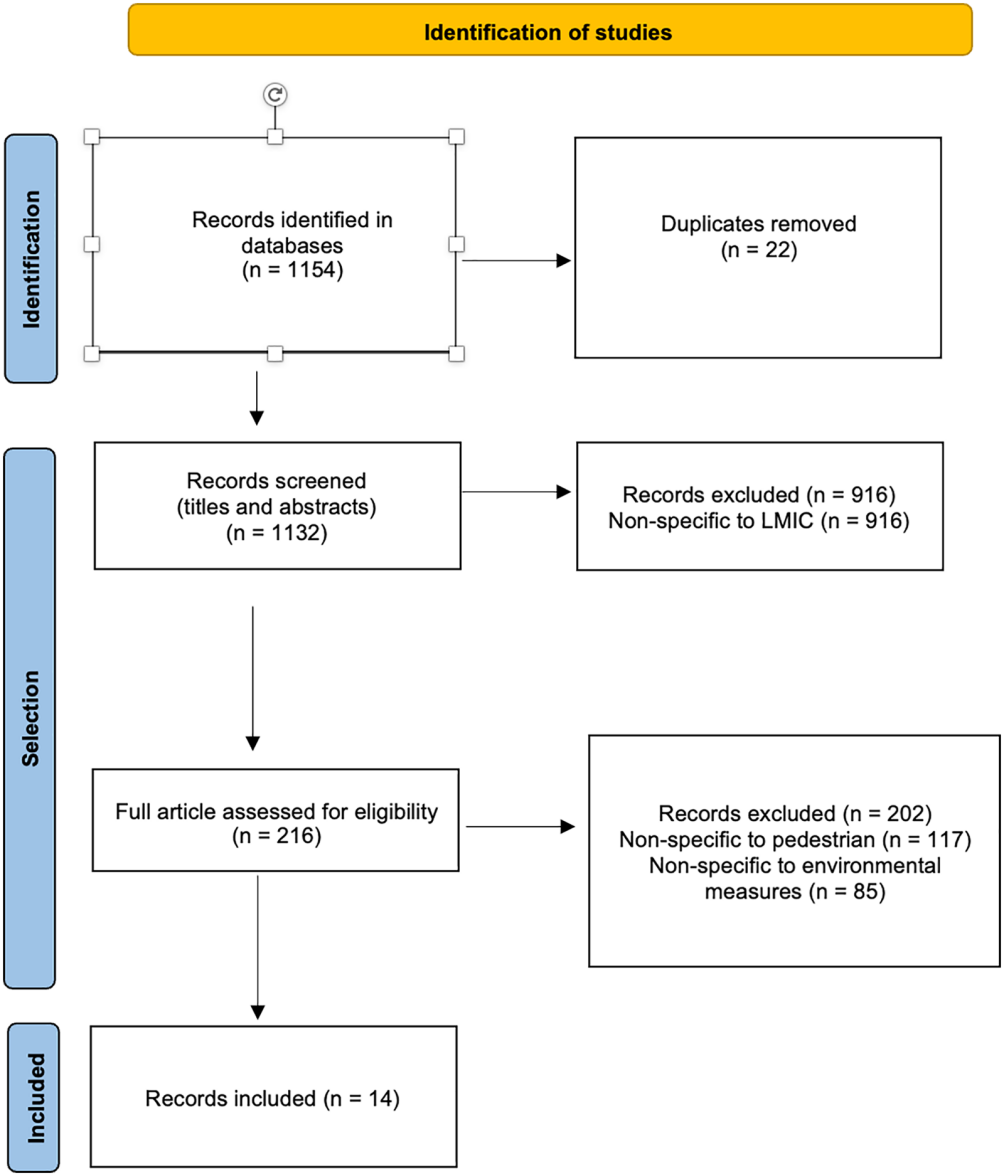
PRISMA flowchart for identification and selection of articles. LMIC, low- and middle-income countries.

All types of studies were considered in this review, regardless of methodology (quantitative, qualitative, mixed), design (experimental, descriptive, etc.), or format (peer-reviewed article, scientific presentation, case study, research report, etc.). All references were exported to Zotero, where duplicates were removed. Titles and abstracts were screened systematically, and any record that did not mention one or several LMIC(s) was removed (see Appendix 2). In a second stage, the articles were read entirely and two other exclusion criteria were applied: written in a language other than French or English; and not presenting original, empirical results. Lastly, only studies that focused on VPIs and environmental measures were retained for the review. The screening process was performed independently by two researchers; in case of a disagreement, a third researcher read the article in full and decided whether to include it or not. A PRISMA flow chart summarizes the number of records at each step of the screening process (Figure 1).

### Data extraction and analysis

Since this was a scoping review, the quality of the included studies was not estimated based on a grading scale, as recommended elsewhere (18). Summary data were extracted using a matrix that specified the first author’s name, the country of study, the study design, the intervention under study, and the study’s main results. A descriptive qualitative analysis was carried out to examine and organize the results according to a thematic framework developed by the WHO (19) that has identified four main categories of environmental interventions to reduce VPIs: (1) traffic calming measures, which include interventions that reduce speed of motorists; (2) measures to reduce pedestrian exposure to vehicular traffic, including road improvements to better separate the different categories of road users or to reduce traffic volume; (3) measures to improve visibility for both pedestrians and motorists; and (4) improvements to vehicle design to maximize pedestrian protection, notably by promoting pedestrian safety assessments and other technical regulations or features.

## Results

After consulting the three databases, 1154 articles were identified, of which 22 duplicates were identified and removed. Of these records, 14 articles dealing with environmental measures implemented in LMICs to reduce VPI risk were retained for the synthesis (Figure 1).

Among the 14 studies, 5 were carried out in Africa (South Africa and Ghana), 4 in Asia (India, Malaysia and Pakistan), and 5 in Latin America (Peru and Colombia). Most studies (12/14) used quantitative data only; the remaining 2 were mixed methods studies. Study designs included pre–post intervention, interrupted time series, case-control, and case study, but no randomized controlled trials met the inclusion criteria. All studies were carried out between 2003–2022 and focused on one of the environmental interventions identified by the WHO, although no study specifically considered improvements in vehicle design (Table 1). A visual representation of the main road features or interventions considered in the 14 studies is provided in Appendix 3.

**Table 1.** Description of the studies included in the review.

Authors (Year)	City, country	Type of study	Intervention/feature under study	Main results and effectiveness of interventions
Afukaar (2003)	Accra and Kumasi, Ghana	Pre–post intervention study	Rumble strip	Installing rumble strips on the main Accra-Kumasi freeway reduced accident CIPs and fatalities
Ahmed *et al.* (2021)	Putrajaya, Malaysia	Descriptive mixed methods study	Zebra crossing	(1) The zebra crossing was the most important to ensure pedestrian safety (2) The four interventions with the most significant impact were: speed limits, crosswalk provisions (time, width, and length of crosswalk), road signs and pedestrian traffic lights
Aidoo *et al.* (2013)	Accra, Ghana	Risk factor analysis Cross-sectional study	Street lightning Speed bumps	(1) In a pedestrian collision, hit-and-run accidents are favored by poor road lighting and weather conditions, poor road conditions, lack of a central median, etc. (2) The use of median separations, speed bumps and the installation of public lighting in road design and construction helps to reduce the risk of VPI and hit-and-run
Cantillo *et al.* (2015)	Bogota, Colombia	Risk factor analysis Cross-sectional study	Footbridges Crosswalks controlled by traffic lights	(1) Pedestrians’ decision to cross a road, to use a footbridge or a signalized intersection is influenced by variables such as the safety/security and attractiveness of each alternative (2) These variables are, in turn, strongly determined by the individual's socioeconomic characteristics and conditioned by the circumstances of the journey (3) Distance to pedestrian bridges or signalized crosswalks is associated with dangerous crossings
Damsere-Derry *et al.* (2019)	Accra, Ghana	Matched case-control study	Speed control Speed cushions Speed bumps	(1) Average vehicle speeds, the proportion of vehicles exceeding the 50 km/h speed limit, and pedestrian fatalities were significantly lower in towns with traffic calming measures (2) Traffic calming devices, including Berliner cousins, were effective in reducing accidents
Donroe *et al.* (2008)	Lima, Peru	Case-control study	Speed control Curb between pedestrians and others	(1) High traffic volumes and high vehicle speeds are associated with the number of fatalities (2) The risk of collisions involving child pedestrians was increased by a high volume of vehicles, lack of lane demarcation, high vehicle speeds and a high density of street vendors
Khatoon *et al.* (2013)	New Delhi, India	Pre–post intervention study	Grade separator Underpass	(1) Dangerous crossings remain at intersections despite the existence of underpasses (2) Removing pedestrian traffic lights due to the underpass has increased speed variability
Nadesan-Reddy and Knight (2013)	Durban, South Africa	Time-series study	(Extended) speed bumps	Speed bumps are associated with a drop in pedestrian-vehicle severe collisions and fatal pedestrian collisions
Quistberg *et al.* (2014)	Lima, Peru	Case-control study	Crosswalks controlled by traffic lights	(1) Collisions were more frequent in the presence of pedestrian signals (2) Longer duration of the pedestrian signal was associated with a higher risk of collision (3) Signals were associated with an increased risk of pedestrian-vehicle collision (4) The presence of a police officer was associated with a reduction in pedestrian collisions
Quistberg *et al.* (2015)	Lima, Peru	Case-control study	Sidewalk Curb between pedestrians and others Chicanes/Barricade	(1) VPIs were less likely in the presence of a curb and sidewalk on both sides of the road and a pedestrian barricade (2) The risk of collision was higher in the presence of street vendors and of parked vehicles 3) VPIs were less frequent in the presence of a chicane or barricade
Goel (2021)	New Delhi, India	Meta-analysis of four cross-sectional studies	Speed reduction	(1) Small reductions in average speed translate into significant reductions in pedestrian fatalities
Umair *et al.* (2022)	Punjab, Pakistan	Risk factor and spatial analysis Cross-sectional study	Green belts and central open areas/islands Narrowing/raised medians Billboards at crossroads	(1) Central areas reduce the number of VPIs, as pedestrians cross the road more safely in two stages (2) Billboards at crossroads increase the occurrence of accidents and distract the drivers (3) Billboards and buildings over three storeys reduce motorists and pedestrians' visibility
Vergel-Tovar *et al.* (2020)	Bogota, Colombia	Risk factor analysis Cross-sectional study	Footbridges Road narrowing/diet	(1) The presence of pedestrian bridges is positively associated with the number of road accidents for all road users (2) Lane width, number of lanes, longer crossing distances for pedestrians, and speeding are positively associated with a high probability of VPI (3) Density and distance to intersections are correlated with road safety
Sinclair and Zuidgeest (2015)	Cape Town, South Africa	Descriptive Cross-sectional study	Footbridges	(1) Pedestrians cross where it suits them best, not necessarily where it’s safest or where there are crosswalks (2) The perception of danger and crime-related activities in/around footbridges influence pedestrian’s decisions to use them, alongside common factors such as convenience, time savings, and perceived traffic risks (3) Jaywalking and crossings on the street occur near bridges

CIP, casualty incident profiles; VPI, vehicle–pedestrian injuries.

### Traffic calming measures

Three different groups of interventions were found in this category: traffic speed reduction measures or speed limits (20–22), vertical deviations, and horizontal deviations (20,23,24). Vertical deviations, like speed bumps, speed cushions, and raised intersections, are rounded, raised features that slow down traffic. Horizontal deviations are typically curb-line arrangements (i.e., chicanes, which require vehicles to follow an S-shaped path) on either side of a two-lane road, road narrowing, or central islands. These measures create a safer and more controlled driving environment, particularly when they are employed together.

Reducing the speed of motor vehicles using environmental measures has great potential to reduce VPIs. In Ghana, the average speed of vehicles and the proportion of motorists exceeding 32 miles per hour (mph) were both statistically lower in areas with traffic calming measures than in control areas, and the risk of VPI was significantly higher in areas without traffic calming measures (odds ratio (OR) = 1.98) (20). In New Delhi, comparing four studies on the impact of speed on pedestrian safety, one study concluded that even small reductions in average speed significantly reduced pedestrian fatalities; a 1% reduction in average speed corresponded to a 7% reduction in pedestrian fatalities (22). The Peruvian study came to similar conclusions: high traffic volumes and high vehicle speeds were associated with a nearly eightfold increase (OR = 7.88) in the risk of mortality and a fivefold (OR = 5.35) increase in the risk of collisions involving child pedestrians (21).

In Colombia, the number of lanes was positively associated with pedestrian casualties (β = 0.982) suggesting that road diet (i.e., reducing the number of travel lanes and/or effective width of the road) can improve pedestrian safety and reduce VPIs (25).

Installing speed humps and extended speed bumps on road sections in two South African districts reduced the number of VPIs in the two areas by 23% (23). The median annual rate of severe collisions between pedestrians and vehicles per kilometer of road also decreased from 1.41 to 0.96 (p = 0.007) and from 2.35 to 1.40 (p < 0.001) in the two zones respectively (23). In Ghana, VPI severity was significantly lower in the presence of traffic calming measures such as speed humps and extended speed bumps. After adjustment for gender, age, and time of day, the risk of pedestrian death was higher (OR = 1.78) for road sections that did not implement traffic calming measures compared with sections that did (20). In towns where traffic calming plans include speed humps, fatal accidents for pedestrians were three times lower. Horizontal detour measures, such as barricades and chicanes, reduce drivers’ field of vision, forcing them to reduce speed and be more attentive to their surroundings. These have been successful in Peru, where VPIs were less frequent in the presence of a chicane or barricade (OR = 0.11) (24).

### Reducing pedestrian exposure

Many LMICs lack pedestrian-specific infrastructure (sidewalks, footpaths), meaning that roads are shared between pedestrians and other road users. As a result, pedestrians are more exposed to the risk of collision than in HICs (26). The literature has identified several road improvements that reduce pedestrians’ exposure to the risk of collisions with vehicles, such as sidewalks, delineations separating pedestrians from road users, zebra crossings, crosswalks controlled by traffic lights, central medians and refuge islands, footbridges, and underpasses (21,25–31).

In Peru, a study showed that VPIs were less likely (OR = 0.19) in the presence of a curb and sidewalk on both sides of the road than in their absence (24). In addition, sidewalks with curbs or barricades were associated with significantly fewer VPI than sidewalks without them. Similarly, the absence of lane demarcations between pedestrians and other road users was associated with a sevenfold increase in the risk of collisions involving child pedestrians (OR = 6.59) (21). In Malaysia, zebra crossings were one of the most effective interventions to improve pedestrian safety, although their effectiveness was conditional on the time required for pedestrians to cross and the length and width of the zebra crossing (32). In Pakistan, medians and refuge islands were associated with a significant reduction in the number of VPIs (β = −116,291; p = 0.038), since they allow pedestrians to cross the road in two stages with a safe space in between (31). In Peru, the presence of a police officer at intersections to regulate traffic considerably reduced the risk of collisions between motor vehicles and pedestrians compared with unsupervised sites (OR = 0.05) (31).

In contrast, some interventions increased rather than decreased the risk of VPIs. Notably, traffic lights at crossroads increased the risk of VPI in Peru (OR = 8.88). Crossing during the pedestrian green signal was associated with a fivefold increase in collision risk for each 15-s increase in crossing time (OR = 5.31) (30). Footbridges and underpasses also showed mixed results, since pedestrians’ decision whether or not to cross a road dangerously does not always depend on the presence of footbridges or underpasses, especially when they do not meet pedestrians’ mobility needs (26). Many other factors, such as the convenience of underpasses and tunnels, their safety, time savings, and the perception of traffic-related risks, affect pedestrians’ willingness to use them (25,28). The proximity of a pedestrian bridge was positively associated with the frequency and lethality of all-type road crashes, although it is unclear whether pedestrians were more at risk or not (25).

Installing rumble strips (road safety features that cause vibration and audible rumbling for motorists) to separate motor vehicles from pedestrians helped limit traffic speed and ultimately reduced the incidence of VPIs by 51% (33).

### Improving pedestrian visibility

A major contributing factor to collisions and fatalities is poor lighting or visibility between pedestrians and other road users. Several studies included in our analysis have described factors affecting pedestrian visibility, how visibility influences safety, and what interventions can be implemented to improve it.

Two studies have suggested that high number and density of street vendors blocking roadways was associated with an increased risk of VPIs (ORs 1.25–2.82 (21,24)), which persisted even when controlling for pedestrian volume. Their location (mostly at street corners) exacerbated the risk since it interfered with visibility of cross-traffic. Reduced visibility because of street vendors was presented as a contributing factor to VPIs, distinct from higher density of pedestrians on the streets. The presence of poorly or illegally parked vehicles was also found to increase the risk of VPI by approximately four times (OR = 3.67) (24). Billboards, which attract road users’ attention and reduce pedestrian visibility, were also associated with a statistically significant increase in VPIs when located near junctions or intersections (β = 174.6771, p = 0.000) (31).

## Discussion

Our scoping review aimed to synthesize evidence on the effectiveness of environmental measures to mitigate the incidence of VPIs in LMICs. While several reviews have examined this topic in HICs, our scoping review found scant data for LMICs. This is especially problematic considering that available evidence indicates that some interventions and features can have heterogeneous effects depending on the context.

This review confirms that traffic calming interventions improve pedestrian safety. This is aligned with previous results from HICs indicating that, for example, the creation of 20 mph traffic zones was effective in reducing motorist speed and improving pedestrian safety (15,34–36). Speed management is not simply a matter of posting regulation signage. Existing speed limits are often disregarded, particularly when authorities have limited capacity to enforce them (19). Our review reveals that to effectively reduce motor vehicle speed, physical and environmental measures must be implemented, such as road narrowing, deviations, speed bumps, or cushions. This is in line with previous evidence gathered in HICs showing that these road features increase drivers’ anticipation of potential threats, enhance their attention and cognitive load, and ultimately lead them to reduce their speed (37–42). Despite their popularity as traffic intervention measures, no studies meeting our inclusion criteria examined the effectiveness of roundabouts to reduce VPI; the effects of these installations on pedestrian safety continue to be a source of debate in HICs (43,44).

There was a paucity of studies on VPIs in rural areas. This may simply reflect the higher incidence of VPIs in cities (45); however, highways between major cities of many LMICs are shared by pedestrians, cyclists, and motor vehicles and give rise to collisions that involve pedestrians. The proportion of fatal injuries shared by pedestrians and motorcycles on rural highways is significantly higher in LMICs than in HICs (36). Lethality for pedestrian victims is 2.3 times higher in rural than urban areas, likely due to higher vehicle speed, inferior road safety infrastructure, and limited access to emergency healthcare (36,46).

Of all the interventions identified in this review, only those designed to reduce pedestrian exposure appear to have mixed effectiveness in LMICs. Traffic lights at pedestrian crossings, footbridges, and underpasses were associated with an increased risk of VPI, in contrast to their protective effect in HICs (47–50). Several hypotheses can explain these conflicting results. When installed at inappropriate crossings (high-speed or multi-lane roads, locations with poor sight distance, and poor compliance with traffic rules), traffic lights can falsely inflate pedestrians’ sense of safety, decrease their vigilance, and even increase their impatience, any of which can ultimately result in increased risk of VPIs (19). Individuals with limited access or with inability, such as wheelchair users or elderly people, may be at even higher risk. Similarly, underpasses and footbridges can increase the distance and effort required to cross a road, which limits their use (26,28). These places are often characterized by (or perceived to be associated with) a high incidence of crime, discouraging their use and nullifying their potential benefits.

Several psychological mechanisms can help clarify this behavior among pedestrians. Waiting time at a signal-controlled crossing is likely to impact the attitude of pedestrians waiting to cross: the longer they have to wait, the greater the risk of crossing on red. Evidence suggests that after a waiting time of 40 s, pedestrians are more inclined to feel impatient and to cross at a red light (35). This impatience can be tempered by systems that discourage pedestrians to cross, such as countdown timers that provide the remaining waiting time for pedestrians at intersections (35). Using the health belief model (i.e., the notion that an individual’s beliefs influence health-related actions or beha-viors), a study revealed that pedestrians were more likely to disregard signals at crossings when (1) they did not perceive any danger of collision, (2) they perceived fewer losses than gains, or (3) they did not have a strong sense of obligation to obey (51). In the same vein, a recent meta-analysis suggests that pedestrians’ decision to cross (or not) was associated mainly with vehicle speed, gap size, and frequency of attempts (52). Their results are in line with those of the present study, and support the recommendations for traffic calming inter-ventions and, to some extent, refuge islands or other midblock street crossings.

While the objective of the present scoping review was focused on pedestrian safety and the effectiveness of interventions, financial considerations cannot be ignored, particularly in contexts with limited resources. Unfortunately, none of the included studies has addressed these aspects. A recent systematic cost–benefit analysis of road safety measures suggests that this ratio can vary significantly between interventions (53). Although the latter analysis was focused on all road users’ safety (not only pedestrians), its results have highlighted that some interventions included in this study were amongst the most cost-effective, namely traffic calming measures (speed humps) or rumble strips.

This scoping review is subject to some limitations. First, its scope was deliberately restricted to studies that examined passive, environmental interventions to reduce VPIs in LMICs. While there are ‘active’ interventions, such as road safety education for motorists and pedestrians, previous systematic reviews have found no evidence of their efficacy (in randomized controlled trials) to reduce injuries (54). This has led some experts to call for a shift in focus from cogni-tive behavioral to environmental interventions in promoting road safety (55), but active and passive interventions cannot always be clearly separated; for example, we discovered that a passive feature (traffic lights at crossings) can produce different effects depending on the level of compliance from road users. Although interactions between different measures are likely, the evidence is very limited – only one of the studies considered in this review evaluated the effects of a combination of several interventions. Finally, this review did not take into consideration other types of vulnerable road users, such as cyclists and motorcyclists, despite the grievous and growing mortality burden among them (40,000 and 380,000 deaths annually, respectively) (56).

## Conclusion

Our scoping review highlighted the effectiveness of some environmental interventions to reduce the risk of VPI in LMICs. Our results reveal that traffic calming measures and road improvements to increase visibility, notably at crossings, are promising strategies to reduce pedestrian injuries. Physical road features like barriers or pylons that separate pedestrians from other road users have also been tested successfully. Improving safety at crossings remains a challenge, since some interventions can be counterproductive by creating a false feeling of security or by increasing impatience.

Our scoping review highlights the importance of conducting in-depth studies reviewing the effectiveness of interventions to improve pe-destrian safety in LMICs, to ensure that the interventions adopted are effective and sustainable. This requires a holistic approach involving key stakeholders such as local authorities, urban planners, road designers and local communities to design interventions tailored to local context. Environmental measures must also take into account the specific needs of pedestrians with physical limitations, such as children, the elderly, and other individuals with limited mobility, hearing, and vision.

## Supplemental Material

sj-docx-1-ped-10.1177_17579759241241513 – Supplemental material for Environmental measures to improve pedestrian safety in low- and middle-income countries: a scoping review Supplemental material, sj-docx-1-ped-10.1177_17579759241241513 for Environmental measures to improve pedestrian safety in low- and middle-income countries: a scoping review by Saidou Sabi Boun, Ronaldo Janvier, Rose Eveyoung Jean Marc, Peterline Paul, Rachel Senat, Joseph Adrien Emmanuel Demes, Guillaume Burigusa, Sarah Chaput, Pierre Maurice and Thomas Druetz in Global Health Promotion

sj-docx-2-ped-10.1177_17579759241241513 – Supplemental material for Environmental measures to improve pedestrian safety in low- and middle-income countries: a scoping review Supplemental material, sj-docx-2-ped-10.1177_17579759241241513 for Environmental measures to improve pedestrian safety in low- and middle-income countries: a scoping review by Saidou Sabi Boun, Ronaldo Janvier, Rose Eveyoung Jean Marc, Peterline Paul, Rachel Senat, Joseph Adrien Emmanuel Demes, Guillaume Burigusa, Sarah Chaput, Pierre Maurice and Thomas Druetz in Global Health Promotion

sj-docx-3-ped-10.1177_17579759241241513 – Supplemental material for Environmental measures to improve pedestrian safety in low- and middle-income countries: a scoping review Supplemental material, sj-docx-3-ped-10.1177_17579759241241513 for Environmental measures to improve pedestrian safety in low- and middle-income countries: a scoping review by Saidou Sabi Boun, Ronaldo Janvier, Rose Eveyoung Jean Marc, Peterline Paul, Rachel Senat, Joseph Adrien Emmanuel Demes, Guillaume Burigusa, Sarah Chaput, Pierre Maurice and Thomas Druetz in Global Health Promotion
