# Supplementary material for: Environmental measures to improve pedestrian safety in low- and middle-income countries: a scoping review
Source: Glob Health Promot. 2024 May 8;31(4):44–55. doi: 10.1177/17579759241241513 (PMC11636016; doi:10.1177/17579759241241513)
Supplement: sj-docx-1-ped-10.1177_17579759241241513 – Supplemental material for Environmental measures to improve pedestrian safety in low- and middle-income countries: a scoping review [file sj-docx-1-ped-10.1177_17579759241241513.docx]

Appendix 1 Definition of keys concepts

| Concepts | Definition |
| --- | --- |
| Road accident | A road accident, also known as a traffic accident, is an unforeseen and undesirable event on the road network involving one or more vehicles that can injure or kill drivers, passengers, pedestrians, or cyclists. According to the Haddon matrix, interactions of three factors allow understanding more of the road accident: environment, human factors, and vehicular in the three phases of the accident before, during and after the accident. |
| Pedestrian | Pedestrians are individuals who travel on foot. They may use a variety of walking aids and devices such as wheelchairs, walkers, canes, skateboards, or roller skates, while carrying objects of various sizes in their hands, on their backs, on their heads, or balanced on their shoulders, or pushing or pulling items. People who run, jog, hike, sit, or lie on the pavement are also classified as pedestrians. |
| Passive environmental measures | Passive road safety measures differ from active measures in that they do not require any participation on the part of the individuals being protected. They involve manipulating technological elements or built environments to mitigate the likelihood of VPIs. |
