# Supplementary material for: Environmental measures to improve pedestrian safety in low- and middle-income countries: a scoping review
Source: Glob Health Promot. 2024 May 8;31(4):44–55. doi: 10.1177/17579759241241513 (PMC11636016; doi:10.1177/17579759241241513)
Supplement: sj-docx-2-ped-10.1177_17579759241241513 – Supplemental material for Environmental measures to improve pedestrian safety in low- and middle-income countries: a scoping review [file sj-docx-2-ped-10.1177_17579759241241513.docx]

Appendix 2 Inclusion and exclusion criteria

| Exclusion criteria stage 1   - The title or abstract does not mention a low-or-middle-income country (LMIC) as the study area. - The article is not written in French nor English. - The article does not present original nor empirical results. |
| --- |
| Inclusion criteria stage 2   - The article focuses on VPIs and passive environmental measures. |
