# Supplementary material for: Environmental measures to improve pedestrian safety in low- and middle-income countries: a scoping review
Source: Glob Health Promot. 2024 May 8;31(4):44–55. doi: 10.1177/17579759241241513 (PMC11636016; doi:10.1177/17579759241241513)
Supplement: sj-docx-3-ped-10.1177_17579759241241513 – Supplemental material for Environmental measures to improve pedestrian safety in low- and middle-income countries: a scoping review [file sj-docx-3-ped-10.1177_17579759241241513.docx]

Appendix 3 Visual representation of environmental measures

1. Rumble Strips

Rumble strips of the road are designed to emit a low-pitched sound when vehicles pass over them. This sound warns drivers to slow down or change direction as they approach a potential obstacle.


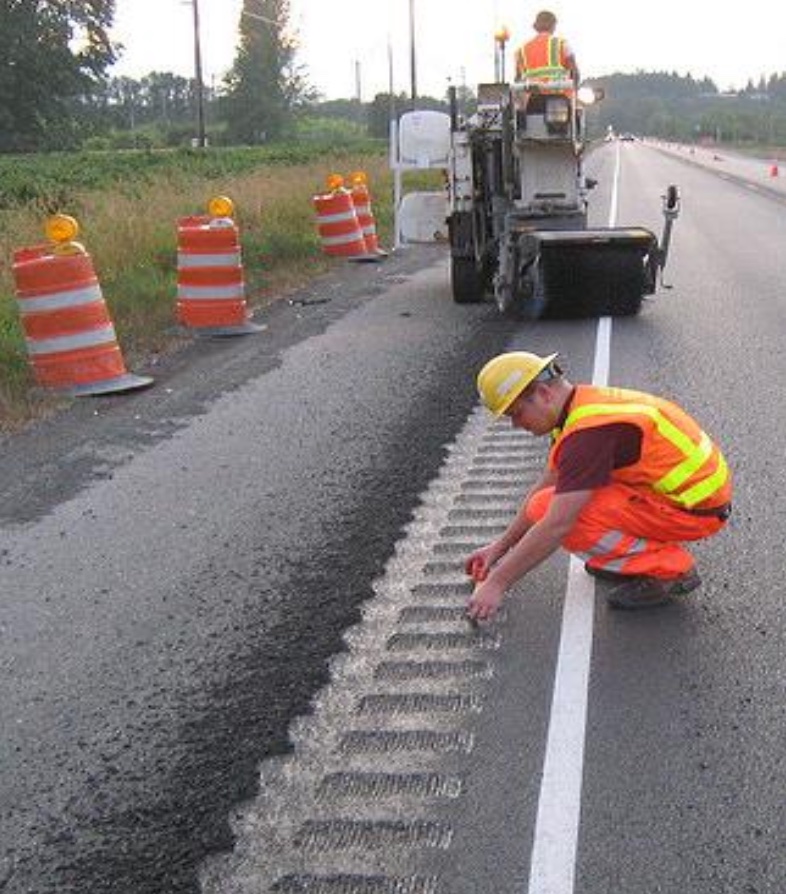


Source : https://doi.org/10.17576/jkukm-2020-32(2)-13

1. Crosswalk, Zebra crosswalk, Zebra crossing

A crosswalk is a device designed to make it easier for pedestrians to cross the road, by delimiting a space with markings indicating that it is shared with pedestrians.


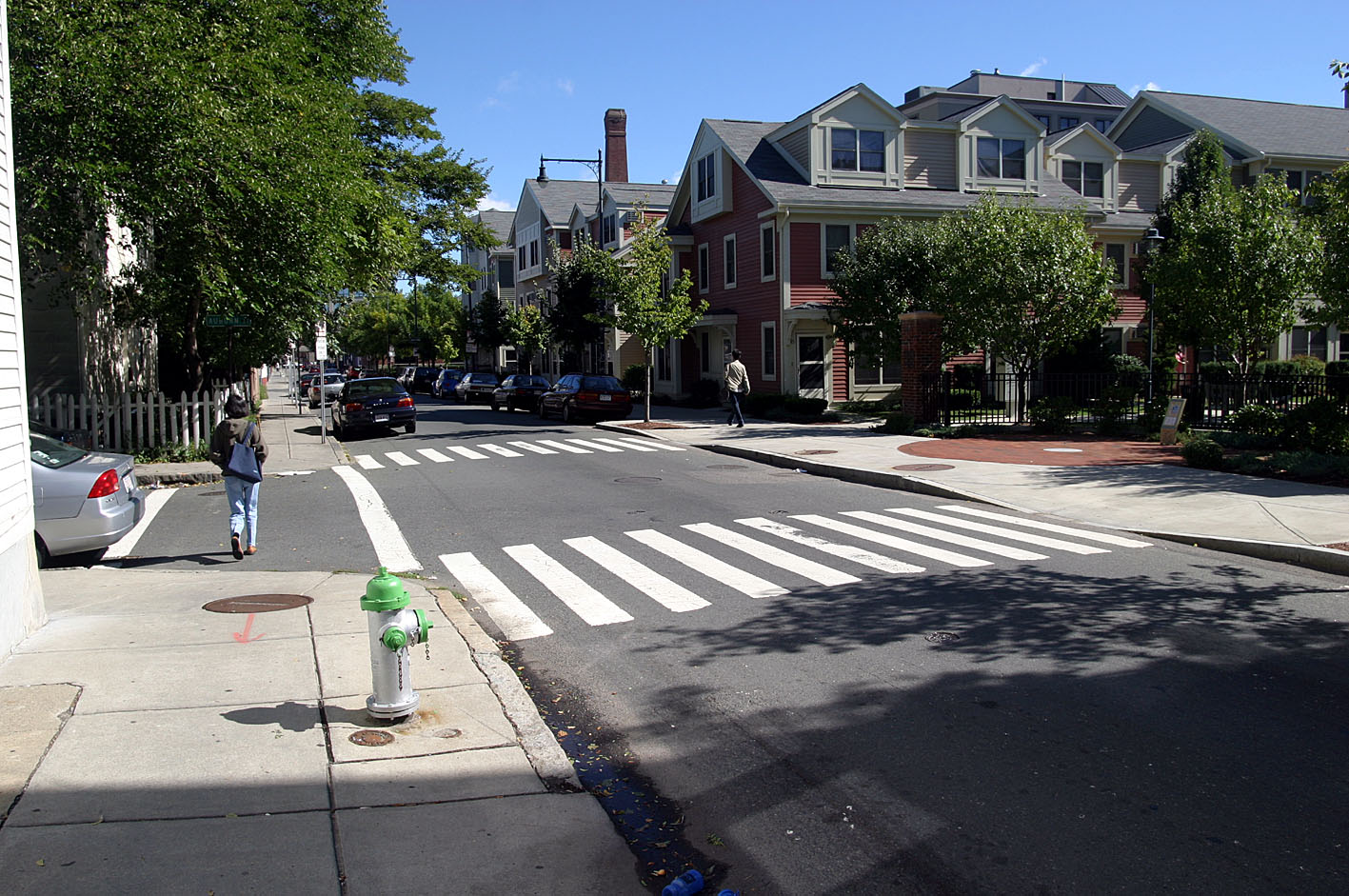


Source: www.pedbikeimages.org

Credit: Dan Burden

1. Footbridges


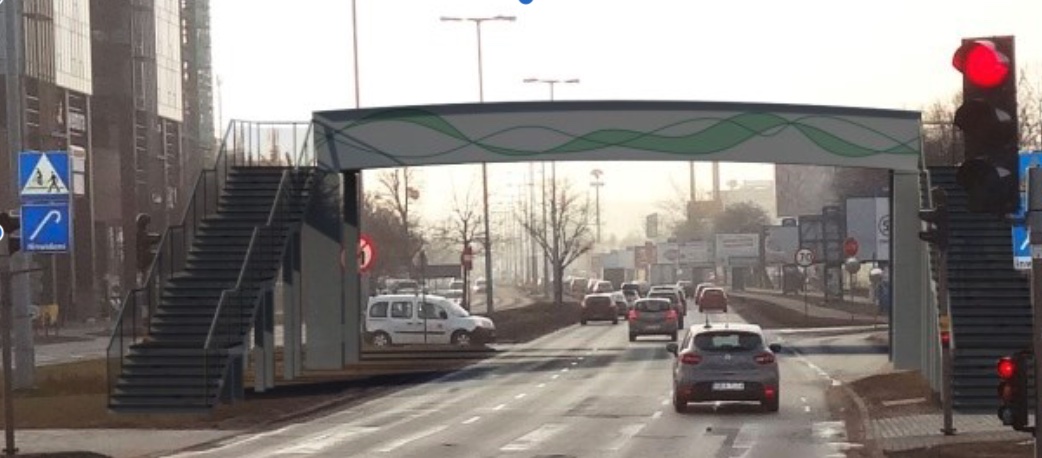


Source : DOI: 10.1051/matecconf/201712201005

1. Chicane/Barricade


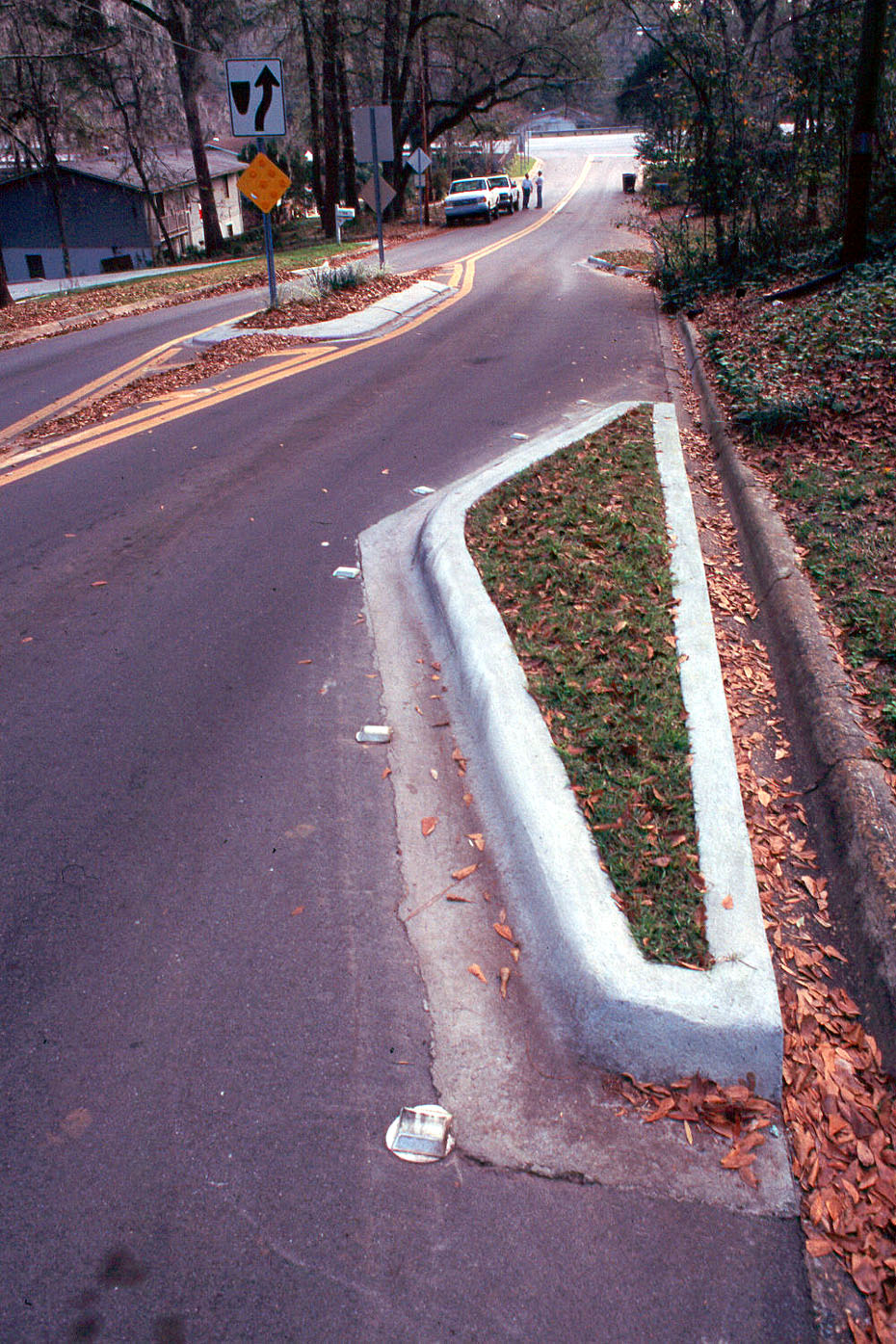
A chicane refers to a sequence of horizontal diversions placed on a straight street to create an "S" shape in the traffic lane. This may include several sidewalk widenings placed alternately to shift traffic horizontally and reduce the road width to one or two narrow lanes, thus requiring motorists to slow down. Low hedges, grass, or tall trees with high crowns can be planted to ensure clear sightlines for drivers and pedestrians.

Source : [www.pedbikeimages.org](http://www.pedbikeimages.org)

Credit: Dan Burden

1. Speed cushions

Cushions are vertical detour designed to act like elongated speed bumps on cars, with minimal effect on heavy vehicles such as emergency vehicles (fire engines, ambulances, etc.) and buses.


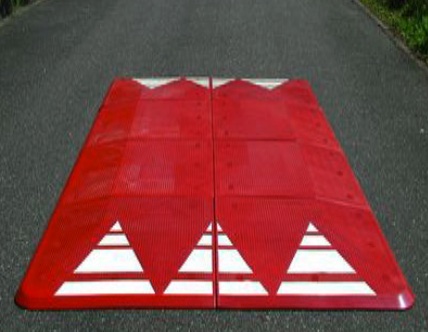


Source: doi:10.2495/UT110591

1. Speed bump

Speed bumps, which should not be confused with speed humps, are narrow vertical deviations typically less than 30 centimetres deep. They can be easily driven over at very low or high speeds, but the suspension can absorb the impact in the latter case. Consequently, they are typically utilized in areas where high speeds are impractical, such as parking lots and alleyways.


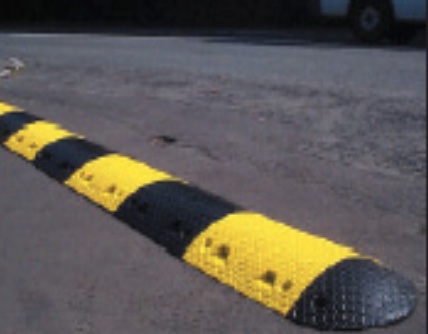


Source : doi:10.2495/UT110591

1. Speed hump

Speed humps, which should not be mistaken for regular speed bumps, are broader and more profound vertical disruptions on the road, measuring three to four meters. These speed humps are designed for low-speed crossings at around 20 mph and are commonly used in residential areas, school zones, parks, and other local streets.


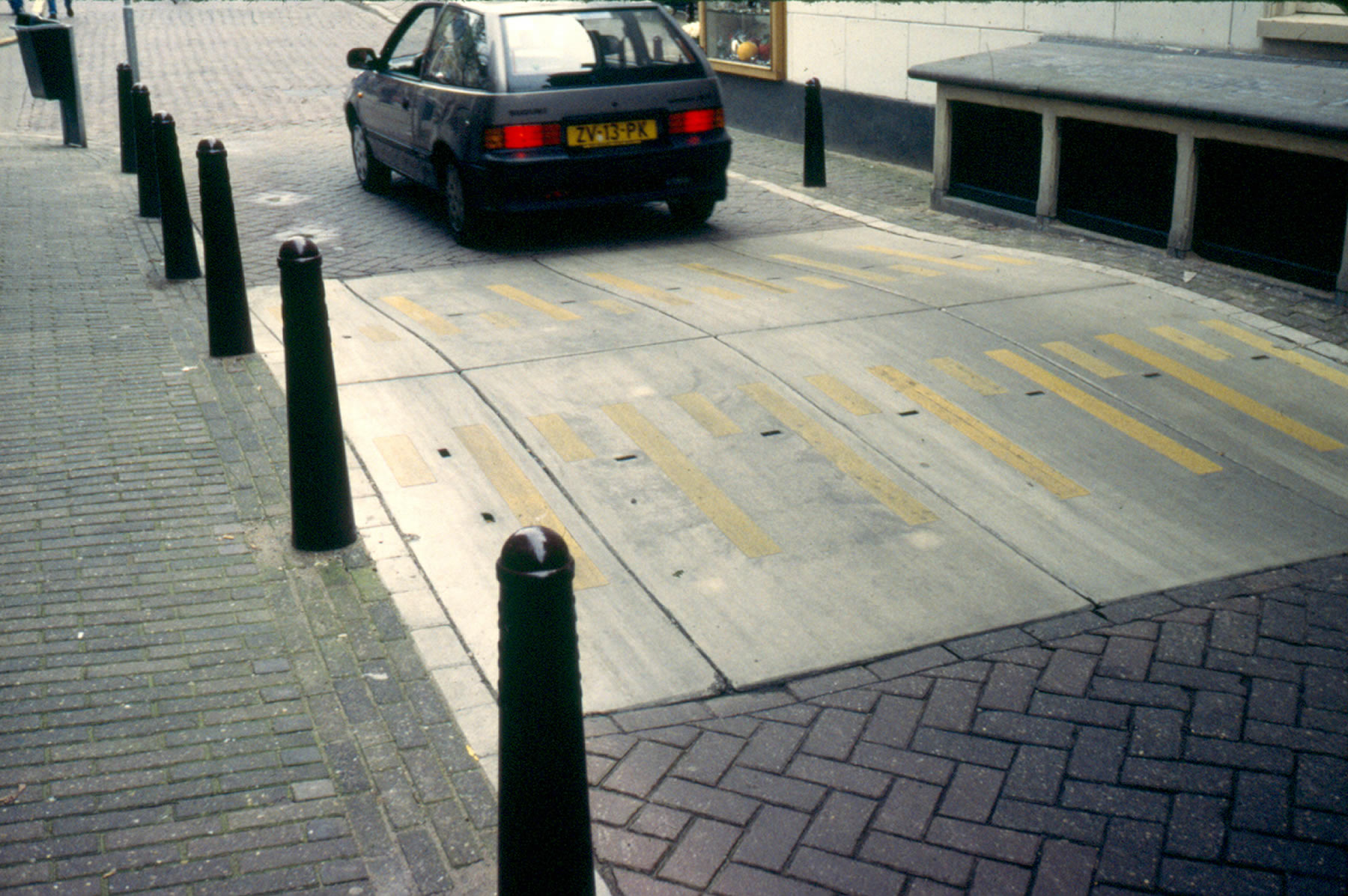


Source: [www.pedbikeimages.org](http://www.pedbikeimages.org)

Credit: Michael King

1. Pedestrian refuge, raised median, Center Island narrowing.

A pedestrian refuge is a median typically located in the center of the street to allow pedestrians to cross in two stages. A median is a raised island typically built on the centerline of two-way streets to separate oncoming traffic and reduce lane width.


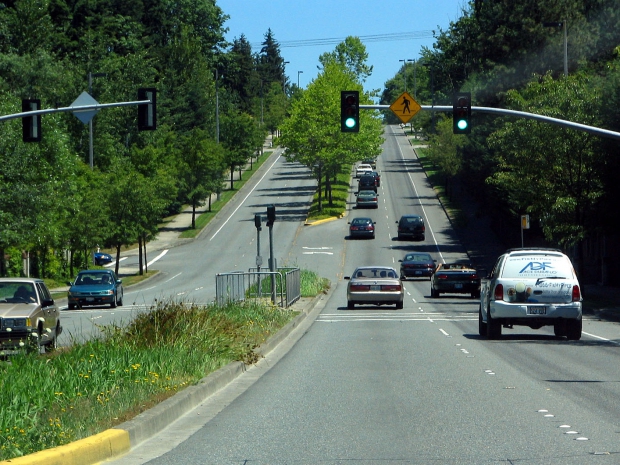


Source: [www.pedbikeimages.org](http://www.pedbikeimages.org)

Credit: Dan Burden

1. Underpass

Underpasses are tunnels that allow continuous pedestrian traffic, separated from car traffic.


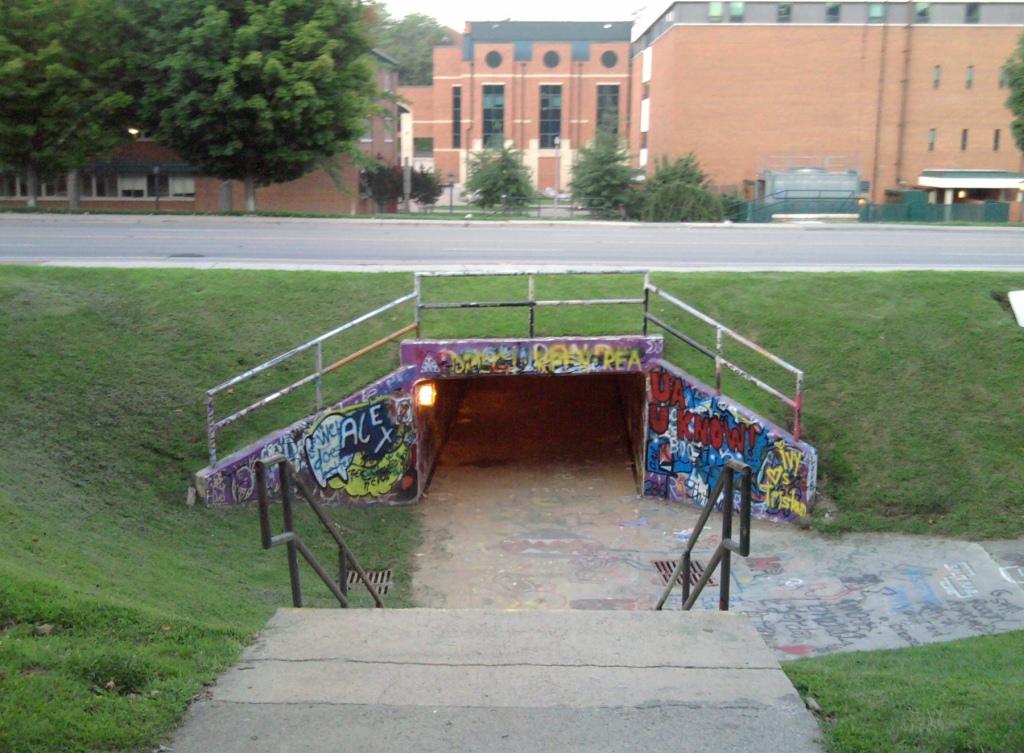


Source : [www.pedbikeimages.org](http://www.pedbikeimages.org)

Credit: Lyubov Zuyevaà

1. Crosswalks controlled by traffic lights.


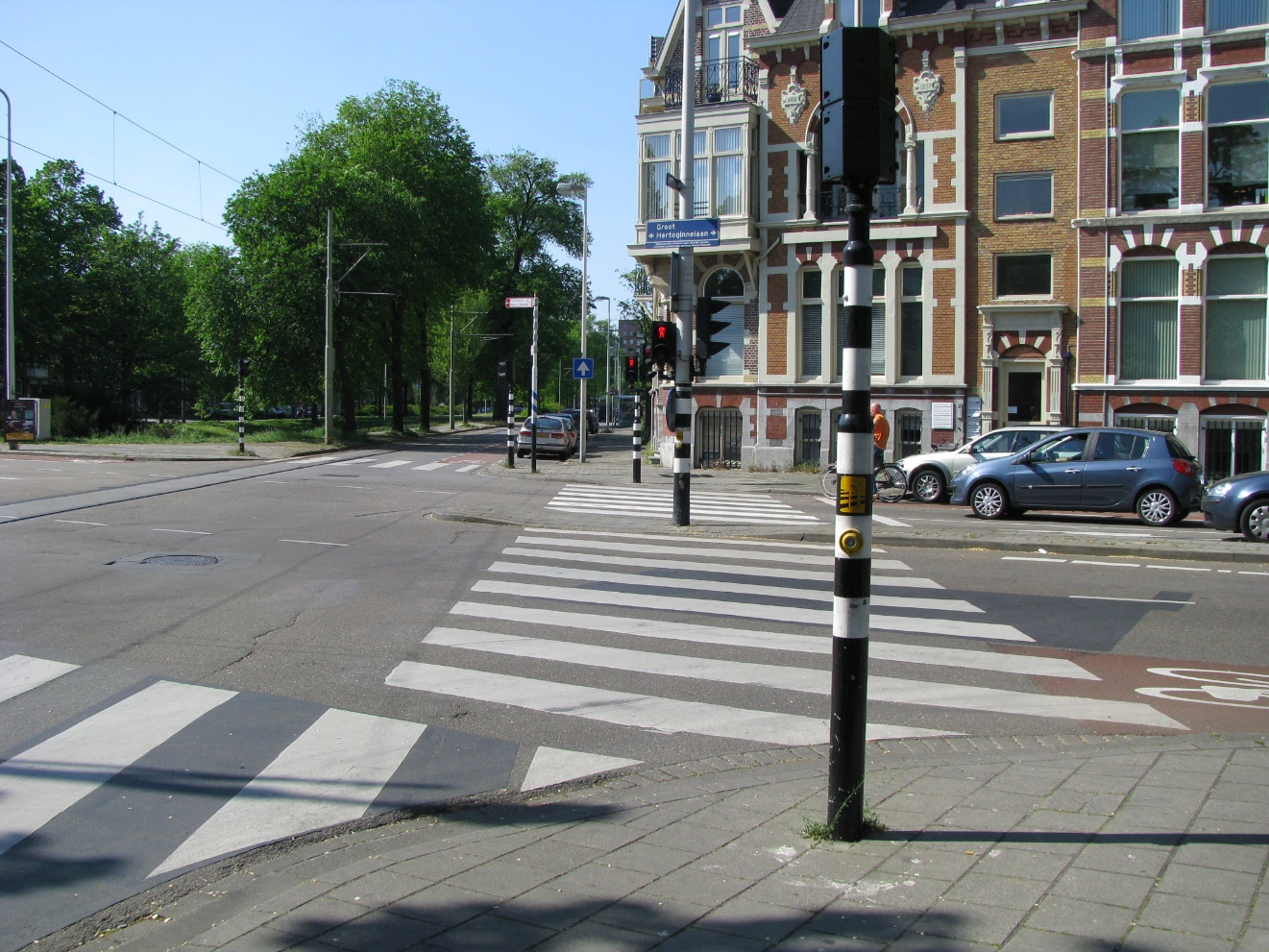


Source : [www.pedbikeimages.org](http://www.pedbikeimages.org)

Photographe: Laura Sandt
